# Are we paying too much attention to cardio-pulmonary nematodes and neglecting old-fashioned worms like *Trichuris vulpis*?

**DOI:** 10.1186/1756-3305-4-32

**Published:** 2011-03-08

**Authors:** Donato Traversa

**Affiliations:** 1 Department of Comparative Biomedical Sciences, University of Teramo, Teramo, Italy

## Abstract

*Trichuris vulpis*, the dog whipworm, causes an intestinal parasitosis of relevance in current canine veterinary practice. Its occurrence is well-known in pets, kennelled dogs and stray animals, and its eggs contaminate the ground in urban areas all over the world. Moreover, *T. vulpis *has been occasionally incriminated, though not convincingly substantiated, as a cause of zoonosis. This nematode is erroneously considered an "old-fashioned" pathogen with a consequent lack of up- to- date knowledge on several aspects of the infection. These, in turn, are still controversial and need to be studied in greater depth. This article reviews current knowledge of *T. vulpis*, together with a discussion of critical points in epidemiology, zoonotic hazard, diagnosis and treatment of canine trichurosis.

## Background

The genus *Trichuris *encompasses nematodes affecting pets, livestock and other hosts, including human beings. These parasites are called whipworms, having a really thick ("whip handle") posterior part of the body and a long and slender anterior part. A mistake in the naming of the genus reflects the destiny of these elusive and enigmatic parasites, *Trichuris*, in fact, means "hair-tail", and was, most likely based on an erroneous past definition that the anterior extremity of the parasite body was the tail and not the cephalic end. An alternative name, i.e. *Trichocephalus*, was thereafter proposed to indicate a "hair-head"-like parasite, but was not accepted by the International Code of Zoological Nomenclature. Consequently, whipworms are classified in the genus *Trichuris *spp., but some authors still call them *Trichocephalus *spp., albeit erroneously [1].

*Trichuris vulpis*, the canine whipworm, and *Trichuris trichiura*, the human whipworm, are the most important species in veterinary and human medicine. Although dogs and canids in general are preferential hosts of *T. vulpis*, its significance for human medicine is its controversial zoonotic ability. The present lack of an update on canine trichurosis is probably due to the fallacy that all major aspects of *T. vulpis *are well known and that there is nothing really new to be investigated or disseminated.

Therefore, the aim of this article is to provide an overview of the state-of-the-art on the most important features of canine whipworm infection, together with a critical discussion of any weaknesses or gaps in our current knowledge. In fact, several moot issues arise if one analyses even the most recent literature available, especially in relation to distribution, diagnostic approaches, zoonotic potential and focused control programs.

## The parasite

**Figure 1:**
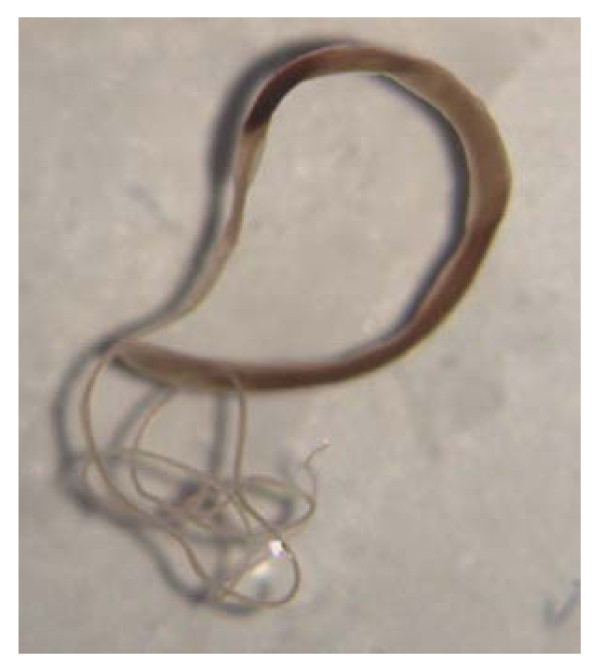
***Trichuris vulpis *adult specimen**.

Adult *T. vulpis *inhabit the large intestine of domestic and wild canids, e.g. dogs and foxes. They are worms a few cm long (~4.5-7.5 cm) in which the thick, broad tail is about one quarter of the total length of the body (Figure 1). The nematodes live with the long and filamentous cephalic end embedded in the mucosa of the caecum and colon of the infected host, while the posterior extremity lies free in the lumen [2,3]. After mating, the females release trichuroid eggs containing a single cell which reach the environment *via *the faeces and, depending on moisture and temperature conditions, embryonate in the soil over a period of three-eight weeks to form an infectious larva inside the eggs. After the infective eggs have been swallowed by a suitable host, the egg plugs are lysed, the larvae hatch and then penetrate the intestinal glands for up to two weeks where they molt before colonizing the large intestine and reaching adulthood. The period of prepatency is about 8-12 weeks [3,4].

The eggs may remain viable and infective in the environment for years, leading to high infection rates in dogs and consequent difficulties in controlling canine trichurosis. More specifically, *T. vulpis *eggs may survive from cold winter to hot summer, especially in wet and shady areas. Unless exposed to extreme conditions for long periods, desiccation and sunlight often do not affect egg viability. Eggs are a constant source of (re-)infection for dogs living in contaminated environments, thus the incidence of trichurosis is higher in adult dogs, which also have higher parasitic burdens than younger animals [3,5-8]. The prevalence rates of intestinal trichurosis in adult dogs may also be due to an absence of a transplacental and/or transmammary transmission in *T. vulpis*, to its long pre-patent period, and to a likely inability to elicit a protective immune response [3,6].

From biological and epidemiological standpoints, a possible association between the presence of *T. vulpis *and of the hookworm *Ancylostoma caninum *has also been detected in infected dogs [6,9,10]. Interestingly, the same association has also been found between human whipworms and hookworms [11]. These findings warrant further studies to understand whether there are biological factors influencing such co-existence or, conversely, whether these associations are only due to overlapping transmission occurring in particular epidemiological settings for susceptible hosts. In general, an awareness of mixed infection is essential to evaluate dogs requiring combined drug treatment, but this is not generally the case for whipworms and hookworms, there being some anthelmintics, notably the benzimidazoles and macrocyclic lactones, which are effective against both *T. vulpis *and ancylostomatids as well. On the other hand, given that some macrocyclic lactones used to treat dog trichurosis have different degrees of efficacy against extra-intestinal nematodes (e.g. *Angiostrongylus vasorum *and *Dirofilaria immitis*) presently emerging in several parts of the world [12], it would be worth evaluating epidemiological patterns between *T. vulpis *and canine cardio-pulmonary nematodes. In fact, repeated drug administration is required to control intestinal trichurosis and such an approach would also simultaneously aid controlling and preventing infections by *A. vasorum *and *D. immitis *(see below).

## Zoonotic or not zoonotic, this is the question

The zoonotic potential of *T. vulpis *is being debated. In the past *T. vulpis *has been reported to cause *visceral larva migrans *(VLM) syndrome and patent intestinal infections in humans [13-15], although dog whipworms are generally not included in zoonotic pet intestinal nematodes [16]. Overall, few cases of presumed human infection by *T. vulpis *have been described (see [17,18]). However, some key examples are discussed below.

The first report of *T. vulpis *infection in humans came when a fragmented female nematode and trichuroid-like eggs were found in the faeces of a child in the USA. The infection was correlated to *T. vulpis *on the basis of the genitalia of the partial parasitic body and the size of the eggs, which were considered by the authors "too large to agree with measurements for eggs of *Trichuris trichiura*" [13]. Indeed, the authors made a "tentative" diagnosis of *T. vulpis *infection, since the absence of male adult specimens prevented a definitive identification [13]. Later on, another presumed diagnosis was made on the basis of large eggs and on the morphology of nematodes (i.e. a gravid female and a partial adult male) histopathologically found in an appendix during a necropsy of a cancer patient in the USA [19]. The male nematode found in the appendix lacked caudal papillae and had a long cloaca, considered key features in distinguishing *T. vulpis *from other species of the genus [19,20]. However, no examination of female characters was performed and, moreover, the authors stated that no "further examination of the posterior male portion" was made, even though they identified the parasites to be *T. vulpis *[19]. Other papers described a case-series of about twenty persons from Japan [14] and the report of a woman with chronic diarrhoea from the USA, diagnosed with patent infections by *T. vulpis *on the basis of egg morphology [15]. Patent intestinal infections by *T. vulpis *in humans are in general considered not scientifically sound and have been questioned [17,21]. The majority of diagnosis of *T. vulpis *infection in humans has relied solely on egg measurements, with no thorough morphological or molecular analysis of adult worms. In fact the main barrier is the size differences between the eggs of the two species, those of *T. vulpis *being nearly twice as large as those of the human whipworm [17,22]. Nonetheless, adult females of *T. trichiura *may produce "abnormal", larger, eggs which may be similar in size to those shed by *T. vulpis *[17,23,24]. Therefore, these eggs, regardless of whether detected together with the regular-sized and smaller *T. trichiura *eggs or not, may lead to a wrong diagnosis of human trichurosis caused by *T. vulpis*. In support of this hypothesis are reports of "mixed" infection in humans from the USA, Mexico and India [15,25,26] which might have been caused by *T. trichiura *shedding both regular and abnormal eggs. Worthy of note is that adult worms recovered from patients with presumed *T. vulpis *infection have been identified to be actually *T. trichiura *shedding abnormal eggs and that also *T. vulpis *eggs can be bigger than regular eggs [17,21]. Hence, apart from the old study which identified large trichuroid eggs together with a *T. vulpis*-like female based on vaginal morphology [13], the actual potential of dog whipworms to produce patent intestinal infections in human beings, at the moment, has to be considered uncertain and still to be definitively established [16,17,21,23,24,27].

Another story is told by *T. vulpis *causing VLM syndrome in both children and adults. In the 80's three cases of VLM caused by *T. vulpis *were reported in Japan [28,29]. In the first paper two brothers were referred with fatigue and eosinophilia. The children lived in a house together with a dog shedding *T. vulpis *eggs, which were found in the house dust. A serological diagnosis of *T. vulpis -*caused VLM syndrome was made and, after the administration of thiabendazole, both children recovered and eosinophils and IgE gradually decreased [28]. The involvement of *T. vulpis *in causing these VLM cases was later questioned on various points, i.e. the lack of a visceral migratory route in *T. vulpis*, specificity limitations of used immunological methods, lack of correlation between antibody titers and larval migration, partial efficacy of thiabendazole against *T. vulpis*, clinical consistency with *Toxocara*-caused VLM in the presence of seropositivity to roundworms and, finally, no proof of larvae encysted in viscera or tissues [30]. This last key information, however, was later provided from the case of an old woman hospitalized with no symptoms but with a shadow on a lung X-ray. After biopsy, the resected tissue revealed fragments of a whipworm. Although the patient did not display high IgE level nor eosinophils, she was serologically positive for *T. vulpis*. A diagnosis of a lung mass due to VLM by dog whipworms was therefore declared [29].

In any case, clear data still need to be provided to definitively add this parasite to the causes of human intestinal infections and/or VLM syndromes. Until further definitive evidence is provided, possibly by the unequivocal DNA-based identification of canine whipworm in human samples in the presence of compatible symptoms and in the absence of other causes, *T. vulpis *still cannot be currently included among zoonotic canine parasites.

## Epidemiological impact on animal and public health

*Trichuris vulpis *is globally distributed with a high prevalence. In the past decades a plethora of studies have been carried out in different countries on the epidemiology of canine parasites, which have also provided information on the dissemination of the canine whipworm. The vast majority of data come from general surveys on the presence of intestinal parasites in privately owned, kennelled and stray dogs and in the environment from a huge range of countries (e.g. see [5-8,21,31-59]). However, no extensive epidemiological studies focusing on canine trichurosis have been undertaken in recent years.

Therefore, data available on the distribution of *T. vulpis *in different countries of the World are markedly heterogeneous from a number of points of view involving influencing drivers (e.g. habitat, origin, breed, season, risk of exposure to parasites, lifestyle, age categories, socioeconomic settings). As a consequence, one should be cautious in extrapolating information from one situation to another in evaluating the risk of infection for dogs and, in theory, for humans.

In general the canine whipworm is a ubiquitous parasite, with high rates of infection on kennelled animals, household pets and stray dogs. For example, infection rates for *T. vulpis *in kennelled dogs can be up to 15-30% as, for instance, in the USA, Belgium and Holland [21,31,34], while prevalence in companion dogs may vary from low rates detected in the past in Great Britain and Greece, i.e. ~2.6-3% [44,45] to the more recent data of ~10-30% found in Greece, Argentina, and France [6,33,46]. Levels of infection in private dogs could appear surprising as, in theory, pets live in good sanitary conditions. Household dogs may become infected through their walks in areas of high risk of infection, e.g. public places, parks and playgrounds. In fact, the main culprit of environmental contamination with whipworm eggs may be stray dogs, which can have infection extensities of up to 60% [47-50].

Recent studies have been carried out in Italy to evaluate the occurrence of canine parasites in both cities and animals. For instance, eggs of *T. vulpis *have been found in stool samples collected from public ground (e.g. squares, gardens, areas for dogs) of big cities (i.e. Bari, Naples, Florence, Milan, Turin) and other towns throughout the country [51-56]. The infection rate in private dogs was <10% in some geographically limited areas [52,57,58]. Unpublished results obtained in 2009-2010 from Central Italy (i.e. Abruzzo and Marche regions) showed a prevalence of trichurosis of 10-15% in privately owned dogs and an infection rate from 30 to 60% in kennelled animals [Traversa, unpublished]. Similarly, *T. vulpis *infection has been detected in 27% of kennelled dogs from different shelters and refuges in the Tuscany region of central Italy [59]. Table 1 reports the results of surveys carried out in the last decade in different regions of Italy which provided information on the presence of whipworm eggs in the faeces of dogs and in samples collected on city grounds. In Table 1 whipworm occurrence is shown in relation to the simultaneous presence of major cardio-pulmonary worms of dogs in the same regions, which is of current concern for their emergence in Italy [12].

**Table 1:** Key examples of results from surveys [[51-59]; Traversa, unpublished data] carried out in the last decade in different regions of Italy on the presence (%) of *Trichuris vulpis *eggs in faeces of privately owned dogs (P), in faeces of kenneled dogs (K) and in environmental samples collected on city grounds (E).

Region	Tv			Av	Di
	**P (%)**	**K (%)**	**E (%)**		

*Northern Italy*					

Lombardy	7.9	nd	3.8-9	+*	+
Piedmont	5.5-8	25	4.1	nk	+

*Central Italy*					

Abruzzo/Veneto§	8.3	59.4	nd	+	+
Abruzzo/Marche	15*	60*	nd	+	+
Abruzzo	nd	nd	10	+	+
Marche	nd	nd	20	+	+
Tuscany	nd	27	4.6	+	+

*Southern Italy*					

Apulia	6	nd	3.3	+	+
Campania	nd	nd	10.1	nk	+

*Insular Italy*					

Sardinia	nd	nd	2	nk	+
Sicily	nd	nd	10	nk	+

Presence (+) of *Angiostrongylus vasorum *(Av) and *Dirofilaria immitis *(Di) in the same regions is also reported on the basis of the information published on recent review articles [12,77]. nd: not determined; nk: not known; *unpublished data; §combined data from a survey in central and northern Italy [57].

If one assumes that *T. vulpis *can infect human beings, the potential public hazard may be concrete in cases of close-contact between humans and companion animals and/or with environmental eggs. For instance, animals adopted by families from kennels or refuges could be a possible source of infection for those members, usually children, most often living with the pet [16]. People acquiring puppies or adults from a dog community or a pet shop need to be aware of the possibility that these animals could be infected by zoonotic parasites. However, they can be easily controlled by adequate anthelmintic administration. It has been suggested that pets living in the homes of people with weak immune systems should be regularly screened for major enteric zoonotic pathogens [60]. It is therefore proposed that *T. vulpis *should be included among these parasites until its zoonotic potential is definitively demonstrated or denied.

Regardless of the actual zoonotic impact of *T. vulpis*, this nematode should always be included in those pathogens to be considered as a possible cause of infections in companion dogs. Repeated faecal examination in shelter-acquired animals is necessary as some cases of infections may be evident only after adoption for the long pre-patent period of *T. vulpis*. When acquired from a shop or a kennel, the animal needs to be examined and/or subjected to a worm control program [16] which, for whipworms, should continue for three months after acquisition [2]. Given that adult dogs are more likely to harbor *T. vulpis *and the high probability that animals have of ingesting eggs in the environment, pets must be regularly screened for *T. vulpis *during their life and, if necessary, undergo an appropriate program with effective anthelmintics. Therefore, an accurate knowledge of the peculiar biological and epidemiological features of *T. vulpis *is essential for a strategic and effective use of parasiticides limiting the epidemiological impact of this whipworm in dogs and, possibly, in humans.

## Clinical significance in veterinary medicine

The pathogenic impact of *T. vulpis *in dog patients is controversial, probably due to the fact that some animals may tolerate a high parasitic burden with no evident clinical signs and to the still questioned ability in sucking blood from the host. The belief that *T. vulpis *does not display a high pathogenicity might also be due to a slow development of the parasite in the infected host, sub-clinical features of the infection in some dogs and the occurrence of symptoms before prepatency.

Indeed, *T. vulpis *causes acute or chronic inflammation at the mucosa of the caecum and, sometimes, of the colon [1-3,61]. The nematode tunnels within the mucosa of the large bowel lacerating the tissues with an oral stylet projected through the oral opening. These whipworms move underneath the epithelium looking for blood and fluids and, in turn, the stylet is introduced into vessels to create blood pools which are ingested [1-3,61].

Puppies may suffer from reduced growth rate and wasting and, in general, infected dogs may present immunological alterations, decreased nutritional conversion, and predisposition to secondary pathogens. Damage to the caecum and colon may be particularly severe in young animals, which may be susceptible to infections caused by hundreds or thousands of parasites. Clinically evident cases are characterized by diarrhoeic episodes alternating with periods of passing normal faeces. In heavy infections, there is mucous, watery and often haemorrhagic diarrhoea, or even pure blood, together with weight loss, lethargy and anemia, caused by blood loss from the damage the whipworms create in the intestinal mucosa.

Animals may suffer from bloody colitis and/or diphtheritic caecitis and of thickening, ulcerative and necrotic lesions on the mucosa; severe anemia and dehydration may cause jaundice and lead to the death of the animal [1-3,61,62].

## Diagnostic challenges

Veterinary practitioners and parasitologists may be faced with different diagnostic challenges inherent to canine whipworm infection.

Because larval *T. vulpis *tunnel into the bowel mucosa to continue their development before the long cephalic end of the adult embeds itself into the intestinal walls, the parasites cause damage even before prepatency is reached [3]. This means that symptoms may occur in the absence of eggs in stool samples examined using copromicroscopic methods, thus leading to a high risk of missed diagnosis. Another cause of false negative results with copromicroscopic analysis is intermittent egg shedding. Repeated faecal examinations are required before ruling out canine trichurosis in a differential diagnosis [3,63].

Eggs of *T. vulpis *may be detected through classic faecal flotation with solutions at different specific gravities (s.gr.). As whipworm eggs have 1.15 s. gr., then a solution of more than 1.20-1.35 s.gr (e.g. using zinc sulphate) should be selected to allow flotation; centrifugation of stool samples before the flotation procedure may help in detection [63,64]. The larger the amount of faeces examined and the longer the set-aside time, the higher is the likelihood of detecting *T. vulpis *eggs [63,64].

**Figure 2:**
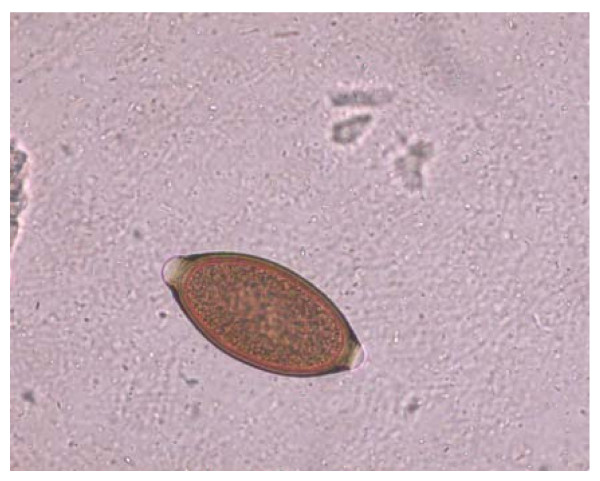
**Floatation with zinc sulphate: *Trichuris vulpis *egg**.

**Figure 3:**
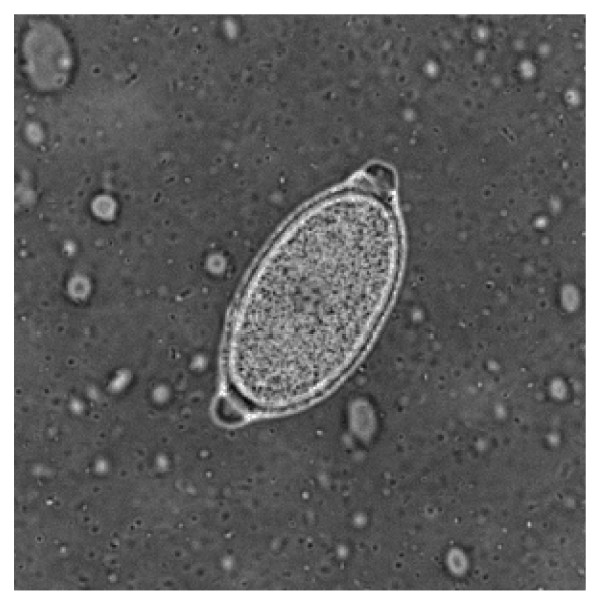
**Floatation with zinc sulphate: *Capillaria aerophila *egg**.

**Figure 4:**
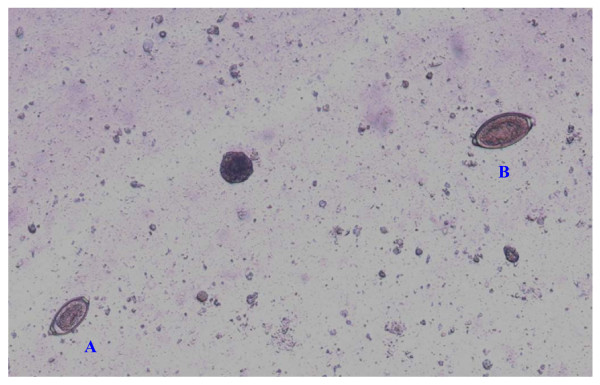
**Floatation with zinc sulphate: mixed infection by *Capillaria aerophila *(A) and *Trichuris vulpis *(B)**.

When barrel-shaped or lemon-like eggs are found, their size, plug aspects and shell wall surface pattern need to be carefully examined to obtain a reliable diagnostic result [12,63] as similar eggs may be shed by other trichuroid nematodes affecting canine patients, e.g. the respiratory *Capillaria aerophila *(syn. *Eucoleus aerophilus*) and *Eucoleus boehmi *[12,63,65,66]. In fact, the close similarity in morphological features of trichuroid eggs makes an accurate identification for microscopists and practitioners very difficult. Eggs of *T. vulpis *are barrel-shaped, brown-yellowish in color, with a thick and smooth shell and two mucoid polar plugs (Figure 2). They measure about 70-90 μm in length and 30-40 μm in width [3,66,67]. Eggs of *Eucoleus *spp. are smaller in both length and width, and have a densely striated wall with a network of anastomosing ridges in *E. aerophilus *(Figure 3) or of tiny pits in *E. boehmi*. Eggs of *E. aerophilus *have asymmetrical bipolar plugs [3,12,66,67]. Hence, of particular importance and of diagnostic significance are mixed infections by *T. vulpis *and *E. aerophilus *(Figure 4), which may often occur [Traversa, unpublished].

A recent study performed on *T. vulpis *isolates from dogs in Spain has provided ribosomal genetic markers instrumental to the molecular identification of the nematode at the species level [68]. Nonetheless, these promising results need to be further corroborated evaluating the usefulness of these findings in biological and epidemiological field studies.

Also, molecular approaches have recently been developed to identify *E. aerophilus *eggs from faeces of naturally infected dogs and cats. The mtDNA-based approach has proved to be promising in further clinical, epidemiological and diagnostic settings into differential diagnosis of Trichuroid-caused infections of pets [Traversa, unpublished].

## Treatment and control: what can we actually do to tackle whipworms in dogs?

The lack of susceptibility of larval and adult stages of *T. vulpis *to some common canine anthelmintics makes the choice of the molecule to be used absolutely crucial.

(Pro-)benzimidazoles are contained in different formulations to be administered to the infected dogs. As an example, a comparative study evaluated the efficacy of three formulations containing mebendazole or fenbendazole alone, or the probenzimidazole febantel in association with other parasiticides [39]. All formulations proved to be effective, although with variable percentages, against infections by whipworms and other common intestinal endoparasites [39].

Experimental and field studies have demonstrated the nearly 100% efficacy of a new anthelmintic tablet formulation containing the cyclooctadepsipeptide emodepside against immature and mature *T. vulpis *and other major canine intestinal nematodes and cestodes [69,70]. Veterinary practices from different EU countries have reported an acceptance rate of 80% of these tablets by treated animals, thus this formulation can be administered with minimal distress to the dog and ease for the owner due to its attractive flavor [69].

Some broad spectrum macrocyclic lactones (e.g. moxidectin, milbemycin oxime) allow for treatment and, possibly, prevention and/or control, not only of *T. vulpis *infection and other intestinal helminthes but also of major extra-intestinal nematodes and ectoparasites. A spot-on formulation available in the USA and the EU containing the endectocide moxidectin 2.5% together with the ectoparasiticide imidacloprid 10% has an efficacy of ~97-100% against *T. vulpis *infection both in dogs with mono-specific infection and in animals infected by ascarids and ancylostomatids at the same time [71,72].

This spot-on formulation also provides efficacy against roundworms and hookworms, treatment and prevention of *A. vasorum *infection, preventative chemoprophylaxis for *D. immitis*, treatment and prevention of fleas, treatment of lice and control of mites.

The efficacy of milbemycin oxime in the removal of whipworms from naturally infected dogs has shown to be ~96-99% [73,74]. This molecule is licensed as an oral formulation for treating and controlling whipworms, roundworms and hookworms, reducing the level of infection by *A. vasorum*, preventing disease by *D. immitis *and, in some EU countries, controlling some canine mites. When associated with the insect growth regulator lufenuron this molecule provides flea prevention and control, as well as treatment and control of intestinal nematodes and prevention of dirofilariosis. If milbemycin oxime is associated with praziquantel the formulation is licensed for the treatment of intestinal nematodes and tapeworms, as well as the prevention of *D. immitis *and the reduction of the level of infection by *A. vasorum*. Oral associations of milbemycin oxime with either lufenuron or praziquantel showed an efficacy of 99.6-100% in treating whipworm infection [69,75].

Key points in controlling *T. vulpis *infection are the long period required by the larvae to reach adulthood and the frequent possibilities of the animals of being re-infected.

The availability of broad-spectrum anthelmintic classes and the belief that a single dose is enough to clear a "generally" infected dog are major causes for negligence in performing diagnostic copromicroscopy in veterinary facilities. In the case of some parasitoses, such as canine trichurosis, the lack of a post-treatment faecal examination could lead to the failure of the control program.

As immatures may resist the activity of parasiticides, for canine intestinal worms with a short prepatent period (e.g. ascarids), a repeated dose within a month is enough to sanitize the dog. Conversely, whipworm larvae take about three months to reach adulthood, so patent infections might sometime recur after administration of a single drug dose after the surviving larvae have matured sexually. Anthelmintic treatments must be repeated at least three times at monthly intervals to kill pre-existing luminal stages and those matured after the first one or two doses [2], thus there is a need for appropriate repeated anthelmintic administration in infected dogs or especially in those animals living in risky areas (e.g. kennelled dogs or pets living in contaminated urban areas) which can repeatedly ingest infective eggs. In support of such considerations a survey carried out in Switzerland demonstrated a 22% yearly incidence of *T. vulpis *infection in dogs treated every 3 months with a broad spectrum anthelmintic formulation containing febantel, pyrantel and praziquantel [37]. In this study, as an inappropriate administration or wrong dosage were considered unlikely, the authors hypothesized that the reoccurrence of *T. vulpis *and other parasite eggs in the faeces of treated animals was probably due to constant re-infections and that the infection risk in privately owned dogs cannot be ruled out despite regular deworming [37].

The European Scientific Counsel Companion Animal Parasites (ESCCAP) recently advised that annual or twice yearly treatments for *Toxocara *spp. does not reduce the risk of patent infections, thus a treatment frequency of at least 4 times per year, or even a monthly worm treatment, is a general recommendation [54,76]. This suggestion might be applied also to whipworm infection considering some features shared by ascarids and whipworms, e.g. geographical spread of the parasites and clinical importance, and especially the high resistance of their eggs in the environment, which greatly favor re-infections throughout the year. When a year-round-control is not performed, regular faecal examinations (e.g. every 1-3 months) of susceptible dogs is a feasible way of evaluating the re-occurrence of trichurosis in previously treated animals.

In the past decade, there has been an increase in the distribution of extra-intestinal nematodes of dogs, e.g. cardiopulmonary *D. immitis *and *A. vasorum *in both endemic areas and previously-free regions of Europe [12,77]. The prevention and/or control of these emerging nematodes requires the monthly administration of macrocyclic lactones to dogs exposed to mosquito bites for *D. immitis *and to ingestion of gastropods for *A. vasorum *(rev. in [12]). Therefore, these molecules may be successfully used against cardiopulmonary nematodes and simultaneously to treat infections with intestinal worms, including *T. vulpis*.

Ivermectin is licensed to prevent canine infection by *D. immitis *but not angiostrongylosis, and to treat infections with hookworms and ascarids, but not with *T. vulpis*. The spot-on formulations containing moxidectin and the oral products containing milbemycin oxime, alone or with other parasiticides, are the most suitable choices against whipworms and other ecto- and endo-parasites, together with the prevention of *D. immitis *(for milbemycine oxime and moxidectin) and *A. vasorum *(moxidectin) infections. Hence, veterinarians have the availability of products licensed to simultaneously prevent cardiopulmonary dirofilariosis (moxidectin and milbemycine oxime), to treat and prevent *A. vasorum *infection (moxidectin) or to reduce the level of infection of angiostrongylosis (milbemycine oxime), to treat and control major intestinal worms and, possibly, to guarantee prevention and/or control of different ectoparasites as well.

In addition to efficacy, the choice of an anthelmintic may be influenced by other features, e.g. ease of administration. While, in general, administration of oral anthelmintics may be difficult in fractious animals or patients that are depressed or moribund [12], the oral formulation containing milbemycin oxime alone showed a 95.5% acceptance rate as a free choice by the animals [78]. While tablets containing milbemycin oxime and praziquantel were reported by different practitioners to be accepted by 64% of treated dogs [69], the recently licensed chewable formulation showed a 94.8% acceptance by animals from the owners' hand [79]. Analogously, the association containing moxidectin and imidacloprid has the advantage of the easy-to-apply dermal spot-on administration in parasitized dogs [80].

**Figure 5:**
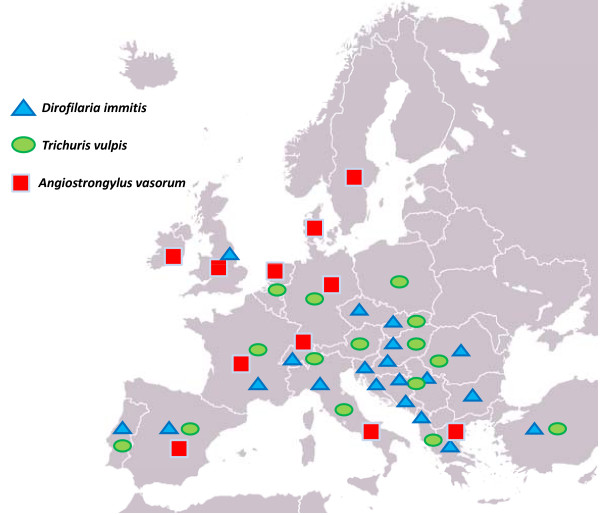
**Map showing key examples on the occurrence of *Trichuris vulpis*, *Dirofilaria immitis *and *Angiostrongylus vasorum *in European countries**.

Especially in North America, there has been some debate on the duration of the monthly chemoprophylaxis against *D. immitis*, i.e. if all year round, six months, or only during the proved season of mosquito activity [2,81,82]. The availability of compounds providing monthly protection from *D. immitis *during the mosquito season and continued control of intestinal infections, could make an interruption of an all year round worm control program undesirable. In fact, in the USA there is a complication in the desire to stop therapy for the dogs of some owners when the risk of heartworm transmission is low or null, considering that dogs may be infected with intestinal nematodes throughout the year [2]. Given that environmental nematode eggs, including those of *T. vulpis*, may remain infective even in the coldest months of the year, there is a rationale to use a broad-spectrum parasiticide all year long to achieve a reliable sanitation of dogs against intestinal and heartworms, regardless of the geographic areas in which this approach may be applied. In the presence of epidemiological risks for nematode infections the availability of broad-spectrum molecules licensed on the market should be perceived not only by veterinarians and owners in the USA, but elsewhere. This is particularly true if one considers that the ectoparasiticides present in some formulations add support to the yearly use of some products in particularly risky situations, given that domestic temperatures usually may allow ectoparasites to survive, develop and infect pets (and, for some ectoparasites, owners), throughout the year [2]. Therefore, in particular epidemiological situations, the all-year round treatment with broad spectrum molecules is advisable to assure treatment, prevention and/or control of major canine parasites. This is of particular importance if one considers that some areas where *T. vulpis *is endemic have recently shown a clear spread of *D. immitis *and *A. vasorum *[12] and now these nematodes are present in several European countries (Figure 5). A key example for Europe is Italy, where *D. immitis *is not only stable in hyper-endemic areas (i.e. Northern regions) where *T. vulpis *has been recently reported in both dogs and in the environment [54,56-58], but also in new autochthonous foci in the south and the center of the Country [12,77,83]. Worthy of note is that *T. vulpis *has been found in urban faecal samples and in kennelled and private dogs from low rates of infection to high values up to 60% [52,57, Traversa unpublished] (Table 1) in central and southern Italian regions where *D. immitis *and *A. vasorum *have recently spread [12].

Other than administrations of an anthelmintic, control of canine trichurosis requires integrated approaches mainly based on hygiene measures.

The high resistance and longevity of *T. vulpis *eggs represent a major barrier for controlling trichurosis, especially when they contaminate soil in shady and damp areas. Dogs being in prolonged contact with contaminated areas and those which encounter, even occasionally, these eggs, tend to be re-infected even after treatment. Therefore, the ultimate goals to be achieved in direct prevention of canine trichurosis are the separation of the animals from the environmental eggs and, if possible, the sanitation of contaminated areas. Appropriate care is required for those public (e.g. parks) or private (kennels, home gardens) areas where infective eggs are protected. These areas need to be thoroughly cleaned. The use of dry or wet heat may help clean contaminated surfaces. Materials such as cement or gravel, mainly in dog runs of kennels or shelters, may help reduce contamination as they provide effective drainage and the absence of soil may help in reducing protection for the eggs. Accurate daily cleaning of drinking bowls and litter boxes in runs and yards of dog communities, and destroying faeces and other waste may also help [1-3,63].

Prompt removal of faeces from places in urban areas were dogs usually defecate is a crucial point, as eggs of *T. vulpis *have been found from soil and faecal samples in public zones from Europe (e.g. [84,85]), especially in several regions of Italy (Table 1), and outside Europe as well, e.g. in USA, Nigeria, Argentina and Brazil [40,86-88]. The removal of dog faeces should be owner-behaviour of all pet owners, and would greatly reduce the incidence of canine trichurosis and other parasitoses in both stray dogs and in companion animals and would also limit the public health hazard for many other parasites as well.

New avenues have recently been opened through natural approaches. A nematophagous fungus recently proved *in vitro *to be a promising biological control agent of *T. vulpis *eggs, although further studies are necessary to evaluate its field ability in reducing environmental contaminations by whipworms [62].

## Concluding remarks

Heterogeneous information on the dissemination of *T. vulpis *in canine populations in Europe as elsewhere, and the lack of updated epidemiological data in the past decade should prompt further studies. Specifically, well-focused epidemiological surveys and new data from privately owned, kennelled and stray dogs, would be pivotal in enhancing knowledge on this canine infection, in understanding the possible risk of transmission from dog to dog (and, maybe, to humans) and, possibly, in preventing any chance of the transmission of canine trichurosis *via *the control of parasitized animals. In fact, studies on even a small or medium scale are central for implementing appropriate control measures in animal and human health. This information would be a key basis towards improved control strategies against (re-)discovered old nematodes (as *T. vulpis*) and presently emerging parasites like *D. immitis *and *A. vasorum*. In fact, it cannot be ruled out that the new favorable epidemiological drivers which are influencing the spreading of extra-intestinal parasitic nematodes [12] could favor the occurrence of more common species and, possibly, new associations in different parasitic compositions.

As an example, past surveys on individuals infected by intestinal nematodes, including the human whipworm, have demonstrated that the distribution of infections is aggregated within populations, with a large number of uninfected individuals and the presence of a small number of "wormy people", who are susceptible to infections with high parasitic burdens [89]. The occurrence of such a predisposition was later confirmed to be a stable feature when analyzing infection levels before and after treatment with parasiticides [90]. Similar studies would also be worth carrying out in canine populations, to evaluate which (if any) are the individual, behavioral, epidemiological and biological drivers predisposing some dogs rather than others to heavy infections with *T. vulpis*. A key point would be the evaluation of the number of drug administrations required in different seasons with the double aim of reducing predisposition to intestinal trichurosis and of preventing infections by cardiopulmonary worms.

Control of canine trichurosis can be a risky challenge for owners and practitioners, especially when epidemiological and biological features of *T. vulpis *are not taken into proper account. Nonetheless, the current availability of products on the market makes this task easier both for a targeted intervention in symptomatic infections as well as for a prolonged worm control program with broad-spectrum anthelmintics. Crucial points influencing every approach are appropriate diagnostic methods, awareness of the local epidemiological situation, knowledge of the risk the animals run of being infected with *T. vulpis *and other parasites, and the compliance of the owner.

In a changing world, with global warming and other drivers (e.g. pet travel, freer trade) influencing parasites' distribution, including zoonotic nematodes affecting dogs, attention should be always maintained high in the control and prevention of "old-fashioned" worms such as *T. vulpis*.

Greater knowledge of the actual zoonotic role of canine whipworms would be an important step in understanding its actual role as a public health hazard. Studies are necessary to unequivocally identify Trichuroid eggs from canine and human faeces in order to clarify the role of dogs as transmitters of *T. trichiura *eggs and the zoonotic significance of *T. vulpis*. *Trichuris vulpis *has been incriminated as a zoonotic nematode but never ultimately demonstrated to be so, while *T. trichiura *has been considered as synonymous with *Trichuris suis *affecting suids [68]. Whether *T. trichiura *and *T. suis *are the same species, thus having the same ability to infect humans, remains to be clarified by further morphological and molecular studies, even if a recent study has evidenced that *T. trichiura *and *T. suis *can be considered to be closely related but genetically different species [91]. Nevertheless, *T. suis *has proved to infect humans and demonstrated to be a possible new, safe, and efficacious alternative for the management of intestinal diseases in humans. It has been hypothesized that human exposure to *T. suis *may afford protection from intestinal immunological diseases, including Crohn's disease, by inhibiting intestinal inflammation [92]. These last considerations confirm that the genus *Trichuris *is less known than we believe. But that is another story.

## Competing interests

The author declares that he has no competing interests.

## Authors' contributions

DT conceived the intellectual content of the article and wrote the text.
